# Aspirin eugenol ester ameliorates LPS-induced inflammatory responses in RAW264.7 cells and mice

**DOI:** 10.3389/fphar.2023.1220780

**Published:** 2023-08-29

**Authors:** Xu Liu, Qi Tao, Youming Shen, Xiwang Liu, Yajun Yang, Ning Ma, Jianyong Li

**Affiliations:** ^1^ Hebei Veterinary Biotechnology Innovation Center, College of Veterinary Medicine, Hebei Agricultural University, Baoding, Hebei, China; ^2^ Key Lab of New Animal Drug Project of Gansu Province, Key Lab of Veterinary Pharmaceutical Development of Ministry of Agriculture and Rural Affairs, Lanzhou Institute of Husbandry and Pharmaceutical Science of Chinese Academy of Agricultural Sciences, Lanzhou, Gansu, China; ^3^ Quality Inspection and Test Center for Fruit and Nursery Stocks, Ministry of Agriculture and Rural Affairs (Xingcheng), Research Institute of Pomology Chinese Academy of Agricultural Sciences, Xingcheng, Liaoning, China

**Keywords:** aspirin eugenol ester, Arachidonic acid, inflammation, UPLC-MS/MS, NF-κB, MAPK

## Abstract

**Introduction:** Inflammation is a defensive response of the body and the pathological basis of many diseases. However, excessive inflammation and chronic inflammation impair the homeostasis of the organism. Arachidonic acid (AA) has a close relationship with inflammation and is the main mediator of the pro-inflammatory response. Based on the prodrug principle, the new pharmaceutical compound aspirin eugenol ester (AEE) was designed and synthesized. However, the effects of AEE on key enzymes, metabolites and inflammatory signaling pathways in the AA metabolic network have not been reported.

**Methods:** In this study, the anti-inflammation effects of AEE were first investigated in mice and RAW264.7 cells in LPS induced inflammation model. Then, the changes of the key enzymes and AA metabolites were explored by RT-PCR and targeted metabolomics. Moreover, the regulatory effects on NF–kB and MAPKS signaling pathways were explored by Western Blotting.

**Results:** Results indicated that AEE significantly reduced the number of leukocyte and increased the lymphocyte percentage. AEE decreased the expression levels of IL-1β, IL-6, IL-8 and TNF-α both *in vivo* and *in vitro*. In the liver of mice, AEE downregulated the levels of AA, prostaglandin D_2_ (PGD_2_) and upregulated 12- hydroxyeicosatetraenoic acid (12-HETE). However, the changes of PGE_2_, PGF_2α_, 6-keto-prostaglandin F_1α_ (6-KETO-PGF_1α_), 9-hydroxy-octadecenoic acid (9- HODE), 13-HODE, 15-HETE, docosahexaenoic acid (DHA) and thromboxane B_2_ (TXB_2_) were not significant. Additionally, it was found that AEE decreased the relative mRNA expression levels of p65 and p38 and the ratio of p-p65/p65.

**Discussion:** It was concluded that AEE might inhibit the LPS-induced inflammatory response through the regulation of AA metabolism. This study provides the theoretical foundation for the development of AEE as a medicinal anti-inflammatory drug.

## 1 Introduction

Inflammation is the most common pathological reaction, which fights infection by releasing proinflammatory cytokines and inflammatory mediators. (Golia et al., 2014). Although the inflammatory response is necessary for keeping the body healthy, when the inflammatory response is too intense, it can cause a large number of tissue rejection reactions and a series of knock-on effects. At present, non-steroidal anti-inflammatory drugs (NSAIDs) and corticosteroids are widely used in the treatment of inflammatory diseases (Lane et al., 2017). Research has revealed that NSAIDs have the advantage of fast onset of action and are more commonly used in clinical practice, but pharmacokinetic and pharmacodynamic tests have shown that these drugs have side effects such as gastrointestinal mucosal damage (Saganuwan and Orinya, 2016). Therefore, the development of new anti-inflammatory treatments is imperative.

Arachidonic acid (AA) is a key constituent of cell membrane lipids. Based on three enzymes, cyclooxygenase (COX), lipoxygenase (LOX) and cytochrome P450 (CYP450), AA can be converted into a variety of metabolites (Miwa et al., 2008; Fan et al., 2015). Prostaglandins (PGs), thromboxane (TX), leukotrienes (LTs) and hydroxyeicosatetraenoic acids (HETEs) are the main metabolites produced by AA and act as major mediators of inflammation (Nakao et al., 1999; Xiao et al., 2016). There is extensive evidence to support that NF-κB and MAPK are two important signal pathways in the process of inflammation. The MAPK and the NF-κB pathways can synergistically contribute to the stimulation of pro-inflammatory cytokine production and release. Therefore, the method of regulating MAPK and NF-κB pathways may have potential in the treatment of inflammation. Targeted metabolomics can be employed to analyze alterations in AA metabolites, facilitating the investigation on the action of mechanism of anti-inflammatory drugs (Zhao et al., 2020). Liquid chromatography-tandem mass spectrometry (LC-MS/MS) combines several analytical techniques to enable qualitative and quantitative analysis of small molecule metabolites. So, the LC-MS/MS platform is employed for comprehensive metabolomic analysis to ascertain overall variations in metabolites (Barton, 2011). Therefore, the study utilized the LC-MS/MS method to identify the alterations in AA metabolites and explore the connection between AA and inflammation.

As we all know, aspirin (Asp), which is the classic drug in the history of medicine, exerts a very significant therapeutic effect on inflammation (Michalska-Małecka et al., 2016). However, the gastrointestinal side effects of Asp limit its application. The primary damage that Asp inflicts on the gastrointestinal tract stems from its irreversible binding with COX enzymes. This binding leads to the reduction of protective prostaglandins of the gastric mucosa, and can ultimately cause the gastrointestinal damage (Rao and Knaus, 2008). In addition, the direct contact between the acidic group (carboxyl group) in the chemical structure of Asp and the gastrointestinal mucosa can lead to gastrointestinal bleeding, constraining the long-term use of Asp (Shen et al., 2019). Eugenol (Eug) is the main component of the volatile oil of lilac flower buds of Myrtle family, which has many pharmacological activities, including antibacterial, antipyretic, anesthetic and analgesic activities. (Fujisawa and Murakami, 2016). It is worth noting that due to the phenolic hydroxyl groups, Eug has strong pungent smell and is unstable because of oxidation that significantly limits.

As a new medicinal compound derived from the esterification of Asp and Eug in line with the prodrug principle, aspirin eugenol ester (AEE) is a white and odorless crystalline solid (Karam et al., 2016). Through the modification of carboxyl and phenolic hydroxyl groups by esterification reaction, AEE not only solves the irritation of the gastrointestinal tract, but also overcomes the instability and irritating odor of Eug. In the body, AEE undergoes catabolism to produce salicylic acid and Eug, both of which act synergistically. The study reported that the LD_50_ of oral administration of AEE in mice was 10.937 g/kg (Li et al., 2012a), which was higher than the LD_50_ of Asp (0.2 g/kg, rats, *p. o.*) and Eug (0.7 g/kg, mice, *p. o.*) (Ichniowski and Hueper, 1946; Ellahueñe et al., 1994), indicating lower toxicity of AEE than Asp and Eug. In rats, after oral administration of AEE (50 mg/kg body weight) for 15 days, no obvious toxicity was detected (Li et al., 2012b). Moreover, the micronucleus test conducted on mouse bone marrow indicated that AEE genetic toxicity was insignificant (Li et al., 2013). Pharmacological investigations have revealed that AEE’s protective effects on vascular endothelial cells by modulating the signaling pathways of nitric oxide synthase (NOS) and nuclear factor-erythroid 2-related factor 2 (Nrf2) and has therapeutic potential in hyperlipidemia and thrombosis (Huang et al., 2019). Previous studies have primarily focused on the pharmacological and toxicological aspects of AEE; further exploration is required to understand its anti-inflammatory properties.

Preliminary experiments were conducted in mice and rats to test the anti-inflammatory, analgesic, and antipyretic effects (Li et al., 2012a). However, the anti-inflammatory mechanism of AEE was unclear and further research is required. Targeting the AA metabolic network and its downstream signaling pathways is a new strategy for the exploration of the action mechanism of anti-inflammation drugs (Pang et al., 2022). In this study, the anti-inflammatory effect of AEE and the difference between Asp and Eug were clarified by the LPS-induced inflammation model. Secondly, the regulatory effects of AEE on AA metabolism were explored through the key enzymes and AA metabolites by RT-PCR and targeted metabolomics, respectively. Finally, the effects of AEE on NF-κB and MAPK signaling pathways were initially analyzed in RAW264.7 cells to investigate possible anti-inflammatory mechanisms. This research provides an experimental basis and foundation for the clinical application of AEE in inflammation-related diseases.

## 2 Materials and methods

### 2.1 Chemical

Mouse macrophages (RAW264.7 cells) were purchased from Shanghai Cell Bank, Chinese Academy of Sciences. AEE (99.5%) was obtained from Lanzhou Institute of Husbandry and Pharmaceutical Sciences of CAAS. (Lanzhou, China). Asp and Eug were supplied by Shanghai Aladdin Biochemical Technology Co., Ltd. (Shanghai, China). Lipopolysaccharide, trypsin, and Cell Counting Kit-8 were from Beijing Solarbio Science & Technology Co., Ltd. (Beijing, China). Foundation FBS and DMEM high glucose medium were purchased from MCE Co., Ltd. (Shanghai, China). ELISA kits including TNF-α (JL10484), IL-1β (JL18442), IL-6 (JL20268) and IL-8 (JL20271) were supplied by Shanghai Jiang Lai Biotechnology Co., Ltd. (Shanghai, China). RNA extraction kit, reverse transcription kit, fluorescence quantitative PCR kit were from Mei5 Biotech Co., Ltd. (Beijing, China). *β*-actin (3700s), p65 (8242s), p-p65 (3033s), p38 (8690s), p-p38 (4511s) were obtained from Cell Signaling Technology., Inc. (Danvers, MA, United States). The isotope internal standards including TXB_2_-d4 (319030), PGE_2_-d9 (10581), PGF_2α_-d4 (316010), 6-keto-PGF_1α_-d4 (15210), PGD_2_-d4 (312010), 9-HODE-d4 (338410), 13-HODE-d4 (338610), 12-HETE-d8 (334570), 15-HETE-d8 (334720), DHA-d5 (10005057), AA-d8 (390010) were purchased from Cayman Chemical Co., (Ann Arbor, MI, United States).

### 2.2 Animal experiment and sample collection

The animal experimental protocol for this study was approved by the Animal Ethics Committee of Hebei Agricultural University (approval No.: 2021106). Fifty-six Kunming mice aged 3 weeks (20–30 g) were provided by SPF Biotechnology Co., Ltd. (Beijing, China). The mice were raised under sufficient lighting, good air conditioning and ventilation, 50% relative humidity and 22°C room temperature. After 1 week of adaptation from purchase, the mice were divided into seven groups (*n* = 8): Control group, Model group, Asp (100 mg/kg) group, Eug (90 mg/kg) group, AEE low (AEEL, 50 mg/kg) group, AEE medium (AEEM, 100 mg/kg) group and AEE high (AEEH, 200 mg/kg) group. AEE, Asp and Eug were mixed thoroughly with 0.5% CMC-Na to prepare the suspension. LPS was dissolved in sterile saline. The mice in the control and model groups received equal volumes of saline, while the mice in the other groups were administrated with the corresponding drugs daily for 7 days. After 30 min of the last administration, the mice were intraperitoneally injected with LPS (1 mg/kg) to induce the inflammation, while the mice in the control group were intraperitoneally injected with saline.

Blood samples were obtained from the orbits of the mice, followed by storage at 4°C for 4 h. Subsequently, the serum was obtained by centrifuging at 3,000 *g* for 10 min to facilitate the detection of inflammatory cytokines. The liver tissue was rapidly frozen in liquid nitrogen and stored at −80°C for subsequent analysis of AA metabolites.

### 2.3 Anti-inflammation effects of AEE in mice

Routine blood analysis with the BC-2800Vet automated blood cell analyze. (Min-dray, Guangzhou, China). The contents of TNF-α, IL-1β, IL-6, and IL-8 were detected by the corresponding ELISA kit (Jiang Lai Bio, Shanghai, China) according to the manufacturer’s instructions.

### 2.4 Determination of AA metabolism levels in liver tissues by UPLC-MS/MS

#### 2.4.1 Extraction of AA metabolites

The liver sample (50 mg) was slowly thawed at 4°C, BHT protein precipitant (500 μL) and 10 μL of internal standard (1 μg/mL) were added, and then homogenized and centrifuged (14000 *g*, 4°C, 10 min). Next, the supernatant (500 μL) was purified to extract AA metabolites by Oasis HLB 96-well plate (Waters, Milford, United States) which was activated by 2 mL methanol and 2 mL water. Then, the plate was washed successively with 2 mL Washing solution A and Washing solution B, and then eluted with 1 mL methanol. The solvent was evaporated by nitrogen. Then, the samples were redissolved in 50 μL acetonitrile/water (1:1, v/v) with 0.1% FA, and centrifuged (14000 *g*, 10 min at 4°C) to obtain the supernatant for UPLC-MS/MS measurements.

#### 2.4.2 UPLC-MS/MS conditions

Samples were analyzed using an ultra-performance liquid chromatography system (Waters UPLC I-Class, United States). The system is equipped with an ACQUITY UPLC BEH C18 column (1.7 μm, 2.1 mm × 50 mm). The mobile phase flow rate was 400 μL/min, the column temperature was 45°C and the sample volume was 4 μL. solvent A was water with 0.1% FA and solvent B was acetonitrile with 0.1% FA. The liquid phase gradients were as follows: 0–1 min, phase B was maintained at 30%; 1–7 min, phase B linearly increased from 30% to 80%; 7–9 min, phase B linearly increased from 80% to 90%; 9–11 min, phase B was held at 90%. To ensure the stability of the assay process, equal amounts of quality control (QC) samples were mixed, and their relative standard deviation (RSD%) was evaluated. The RSD% results for the QC samples can be seen in Supplementary Figure S1. The RSD% for each tested substance was <30% (Muratovic et al., 2015), indicating the reliability and stability of the experimental data.

The Mass Spectrometry (MS) analysis was conducted using a 5500 QTRAP mass spectrometer (AB SCIEX, US) in negative ion mode. The specific parameters used were as follows: a source temperature of 450°C, Ion Source Gas1 (Gas1) set at 55 psi, Ion Source Gas2 (Gas2) set at 60 psi, curtain gas at 30 psi, and Ion spray voltage floating (ISVF) at −4500 V. To measure the ion pair, multiple reaction monitoring (MRM) mode was employed for detection. Signal to noise ratio (S/D) was used to determine the limit of detection (LOD, S/D > 3) and the limit of quantification (LOQ, S/D > 10). The LOD of the AA metabolites were ranged from 0.25–1,000 ng/mL. The results of quantification and confirmation masses, retention time and standard curves were summarized in Supplementary Table S1.

### 2.5 RT-PCR analysis

According to the kit instructions, total RNA was extracted from the liver tissue, and the mRNA was reverse transcribed into cDNA. The cDNA was then utilized in RT-PCR to assess the relative transcription levels of COX-1, COX-2, 5-LOX, PLA2, and CYP450 genes. The PCR primers were provided in Supplementary Table S2, with *β*-actin selected as the internal reference gene. The RT-PCR reaction conditions at the setting of 95°C for 30 s, followed by 40 cycles of 95°C for 5 s, and 60°Cfor 30 s. The relative amount of mRNA transcription was determined using the 2^−△△CT^ method.

### 2.6 Anti-inflammation effects of AEE in RAW264.7 cells

RAW264.7 cells were culturing in DMEM high sugar medium (containing 10% fetal bovine serum and 90% DMEM high sugar medium) in an incubator at 37°C and 5% CO_2_. Asepsis was performed throughout and cell status and growth were observed daily.

Cytotoxicity was assayed by the CCK-8 method according to the instructions. Inoculate 96-well plates with cell suspension (100 μL/well) and place the plates in an incubator for 24 h. Add AEE solution at concentrations (5, 15, 45, 75, 100, 150, 300, 350 μM) and incubate for 24 h. Subsequently, 10 μL of CCK8 solution was added to each well, followed by 2 h incubation in the incubator. Finally, the absorbance at 450 nm was measured using a microplate reader, and the cell survival rate was calculated.

The content of TNF-α, IL-6, IL-8, and IL-1β was measured using the ELISA method. The cells were divided into the following groups, control group (Control): normal culture for 24 h; inflammation model group (Model): 1 μg/mL of LPS for 24 h; LPS + ASP: pre-protected cells by adding 150 μM of ASP to the culture medium for 4 h and 1 μg/mL of LPS for 24 h; LPS + Eug: pre-protected cells by adding 150 μM of Eug to the culture medium for 4 h and 1 μg/mL of LPS for 24 h. Eug for 4 h and 1 μg/mL of LPS for 24 h. LPS + AEE (low, medium and high concentrations): pre-protection of cells by adding 75 μM, 150 μM and 300 μM of AEE to the culture medium for 4 h and 1 μg/mL of LPS for 24 h. The RAW264.7 cells in the logarithmic growth stage were seeded in 48-well plates, and the supernatant from each group of cells was collected after the completion of the culture. The ELISA kit instructions were followed for further analysis.

The cells were cultured in 24-well plates, and after 24 h of culture, total RNA was extracted and analyzed for p65 and p38 content by reverse transcription. PCR primers can be found in Supplementary Table S2, and select *β*-actin as an internal reference gene. Finally, the corresponding RT-PCR kit was used for detection following the manufacturer’s instructions. The reaction conditions for RT-PCR were 95°C for 30 s, 95°C for 5 s, 60°C for 30 s and 40 cycles. The relative transcript amount of mRNA was calculated using the 2^−ΔΔCt^ method.

### 2.7 Western blotting assay

The RAW264.7 cells were treated with media containing different concentrations of AEE (150 μM) for 24 h, followed by 24 h treatment by LPS (1 μg/mL). The lysate was centrifuged at 14,000 *g* for 10 min at 4°C and collected the cell supernatant. The protein concentration was determined by the BSA method and the protein concentration was calculated from the standard curve. The protein was denatured by adding protein loading buffer and boiling at 100°C for 10 min. Gel electrophoresis was carried out and the proteins were transferred to PVDF membrane. The membranes were washed for 5 min in TBST, 3 times, and closed at room temperature for 1 h. The primary antibodies for p65, p-p65, P38, p-p38, and *β*-actin proteins were incubated overnight at 4°C and then incubated with secondary antibodies for 2 h. Protein bands were visualized using an enhanced chemiluminescence kit and detected using a BIO-RAD imaging system (BIO-RAD, Hercules, CA, United States). ImageJ 18.0 software was used to analyze the protein bands for grey-scale values.

### 2.8 Statistical analysis

Statistical analysis was conducted using SPSS version 26.0 (SPSS Inc., Chicago IL, United States). The data were expressed in means ± SD. One-way ANOVA was used for statistical analysis, followed by Tukey’s for *post hoc* comparison. When *p* ≤ 0.05, the difference was statistically significant. The chromatographic peak areas and retention times were extracted using Multi quant software (Multi Quant™, SCIEX, United States). The AA content in the sample was determined based on the standard curve.

## 3 Results

### 3.1 Effects of AEE on blood routine indexes of mice

The results of the routine blood analysis showed a significant decrease in both leukocyte count (WBC) and granulocyte percentage (Gran) in the control group compared to the model group (*p* < 0.05) (Table 1). Conversely, the control group exhibited a significant increase in lymphocyte percentage (LYMPH) than the model group (*p* < 0.01). In comparison with the model group, the WBC in Asp group decreased significantly (*p* < 0.01), while LYMPH percentage in Eug group significantly increased (*p* < 0.05). Additionally, the results of WBC in Eug, AEEL, AEEM and AEEH groups were significant decreased than that in the model group (*p* < 0.05). The results of WBC, RBC, LYMPH, Gran, MCH, RDW, and MPV among AEEL, AEEM and AEEH groups were not significant (*p* > 0.05).

**TABLE 1:** Blood parameters of the AEE on LPS induced inflammation in mice (*n* = 8).

Groups	WBC (10^9^/L)	RBC (10^12^/L)	LYMPH (%)	Gran (%)	MCH (pg)	RDW (%)	MPV (fL)
Control	1.90 ± 0.61^aa^	9.20 ± 1.81	72.9 ± 9.97^aa^	25.5 ± 8.67^aa^	14.6 ± 0.29	13.8 ± 0.006	6.8 ± 0.23
Model	4.10 ± 1.66	9.90 ± 2.47	43.3 ± 6.79	46.6 ± 6.27	14.7 ± 0.57	14.5 ± 0.003	6.9 ± 0.24
Asp	2.20 ± 0.43^aa^	9.40 ± 1.23	60.5 ± 5.49	36.0 ± 5.98	14.7 ± 0.69	13.9 ± 0.003	6.6 ± 0.23
Eug	2.50 ± 0.55^a^	9.70 ± 1.16	55.3 ± 9.14^a^	41.3 ± 10.8	14.3 ± 0.32	14.3 ± 0.006	6.9 ± 0.23
AEEL	2.60 ± 0.69^a^	9.60 ± 1.36	61.5 ± 12.8	36.6 ± 12.3	14.2 ± 0.21	14.0 ± 0.002	7.0 ± 0.28
AEEM	2.80 ± 0.80^a^	10.3 ± 0.64	63.7 ± 8.63	36.1 ± 2.98	14.2 ± 0.55	14.5 ± 0.007	6.6 ± 0.32
AEEH	2.80 ± 1.08^a^	10.2 ± 0.91	59.2 ± 13.9	38.0 ± 11.6	14.4 ± 0.71	14.1 ± 0.002	6.5 ± 0.25

Abbreviation: WBC, leukocyte; RBC, red blood cell; LYMPH, lymphocyte percentage; Gran, granulocyte percentage; MCH, mean corpuscular hemoglobin; RDW, red blood cell distribution width; MPV, mean platelet volume. Compared with model group: ^a^ *P* < 0.05, ^aa^ *P* < 0.01.

### 3.2 Effects of AEE on inflammatory cytokines in mice

**FIGURE 1:**
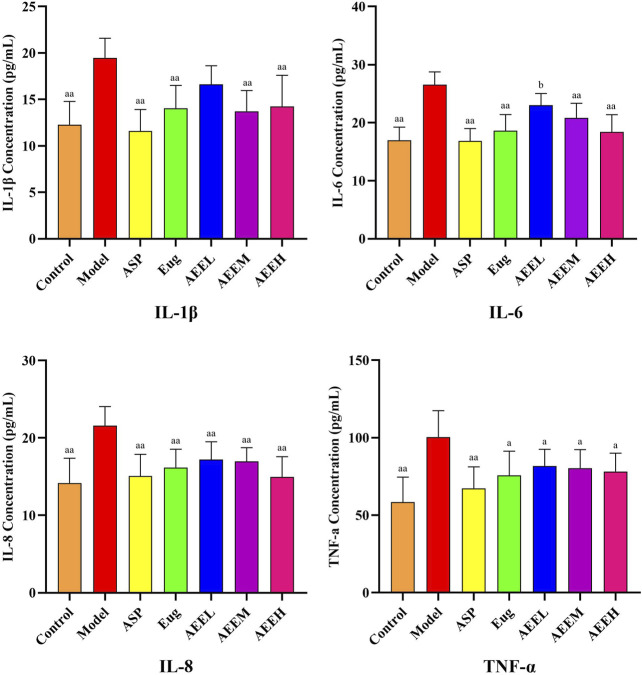
Effect of AEE on IL-1β, IL-6, IL-8, TNF-α induced by LPS in mice (*n* = 8). Compared with model group: ^a^ *P* < 0.05, ^aa^ *P* < 0.01; Compared with Asp group: ^b^ *P* < 0.05.

The levels of inflammatory cytokines IL-1β, IL-6, IL-8, and TNF-α were significantly increased in the mice of the model group compared to the control group (*p* < 0.01) (Figure 1). Regarding to IL-1β and IL-6, no difference was observed between the model and AEEL groups, while the IL-1β and IL-6 levels in Asp, Eug, AEEM and AEEH groups were significantly lower than that in the model group (*p* < 0.01). As compared with the model group, the level of TNF-α in Eug and AEE groups were significantly lower (*p* < 0.05), and Asp treatment also extremely reduced the TNF-α level (*p* < 0.01). Treatment with AEEM significantly decreased the levels of IL-1β, IL-6, and IL-8 (*p* < 0.01). There were no significant differences in the results of IL-1β, IL-6, IL-8 and TNF-α among the AEEM, AEEH, Asp and Eug groups (*p* > 0.05).

### 3.3 Effects of AEE on AA metabolic pathway

**FIGURE 2:**
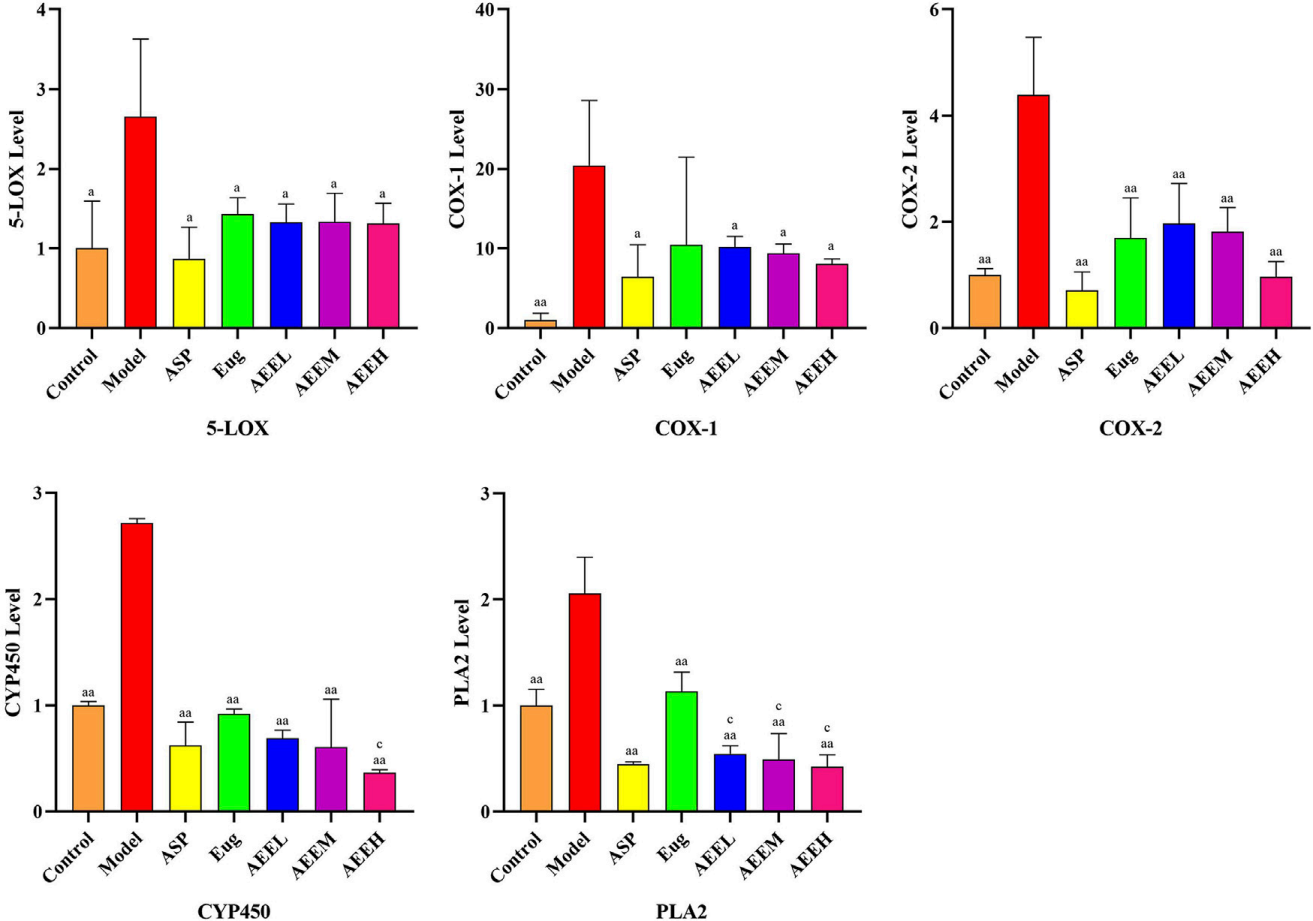
Effects of AEE on 5-LOX, COX-1, COX-2, CYP450 and PLA2 induced by LPS (*n* = 5). Compared with model group: ^a^ *P* < 0.05, ^aa^ *P* < 0.01; Compared with Eug group: ^c^ *P* < 0.05.

We first detected mRNA changes in the enzymes of the AA metabolic pathway. The results revealed a significant increase in the expression of CYP450, PLA2, COX-2, COX-1, and 5-LOX in the model group compared to the control group (*p* < 0.01) (Figure 2). AEE treatment significant decrease in the mRNA expression of CYP450, PLA2, COX-2, COX-1, and 5-LOX (*p* < 0.01). Moreover, the expression of PLA2 was significantly decreased in the AEE group compared to the Eug group (*p* < 0.01). There were no significant differences observed among the different doses of AEE groups (*p* > 0.05).

**FIGURE 3:**
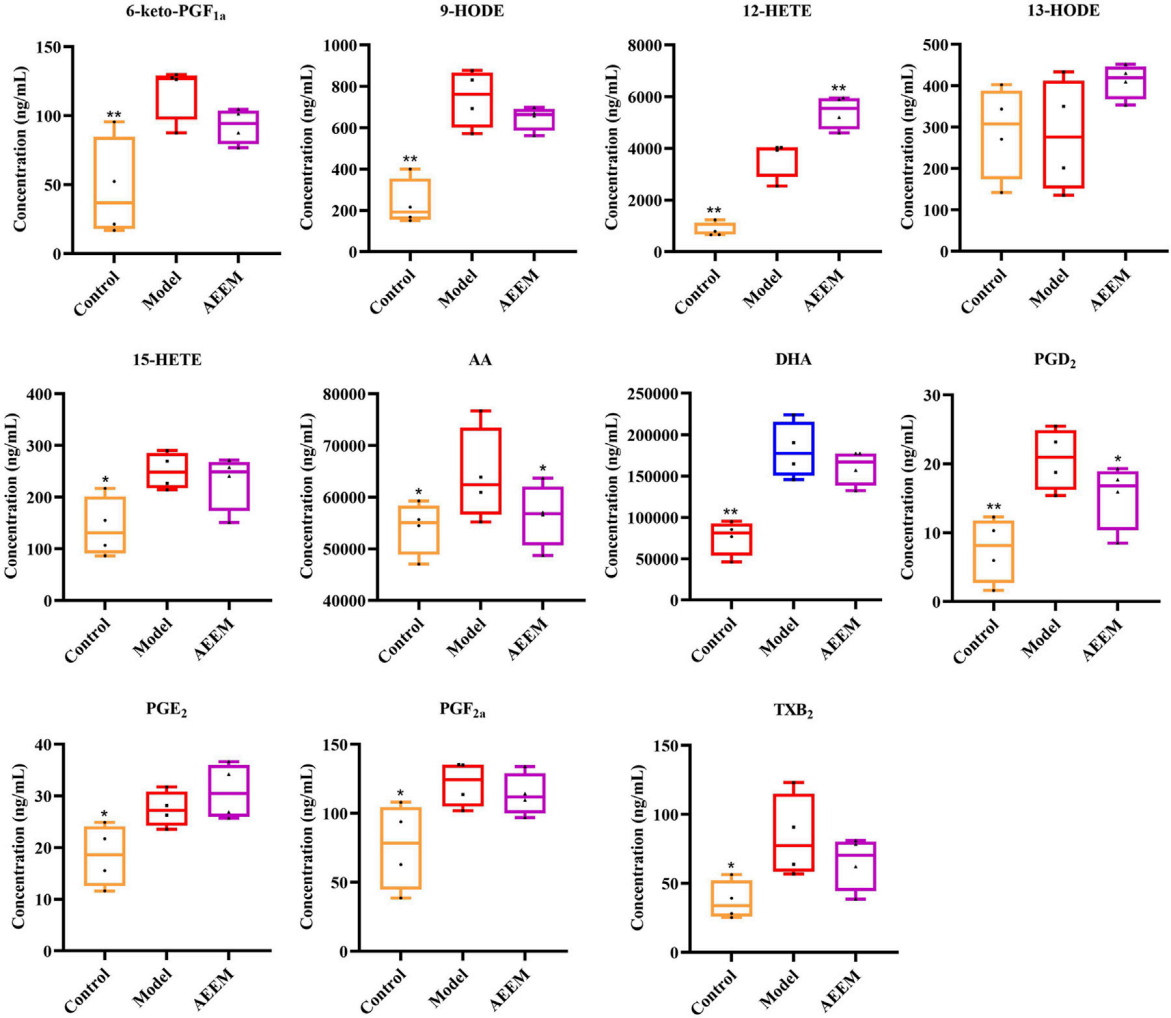
Effect of AEE on the concentration of AA metabolites in the liver (*n* = 4). Compared with model group: ^*^ *p* < 0.05, ^**^ *p* < 0.01.

Based on the findings from the analysis of inflammatory cytokines and AA enzymes, AEEM exhibited a pronounced anti-inflammatory effect. Consequently, four liver samples from the AEEM group were chosen for targeted metabolomics analysis using LC-MS/MS. The results demonstrated a significant decreased in the concentrations of AA and PGD_2_ (*p* < 0.05), while the concentration of 12-hydroxyeicosatetraenoic acid (12-HETE) was significantly increased (*p* < 0.01) in the AEEM group (Figure 3). There was no difference in the concentrations of PGE_2_, 13-hydroxy-octadecenoic acid (13-HODE), 12-HETE, TXB_2_, PGF_2α_, PGD_2_, docosahexaenoic acid (DHA), 15-HETE, 9-HODE and 6-KETO-PGF_1α_ between model and AEEM groups (*p* > 0.05). In Supplementary Figure S2, the heat map colors from red to blue indicate high to low concentrations of metabolites. The color was gradually changing from blue to red between the control group and the model group. Notably, the AEEM and model groups were clustered into one group, but the AEEM and control groups were more closely located (Supplementary Figure S2).

### 3.4 Anti-inflammation effects of AEE in RAW264.7 cells

**FIGURE 4:**
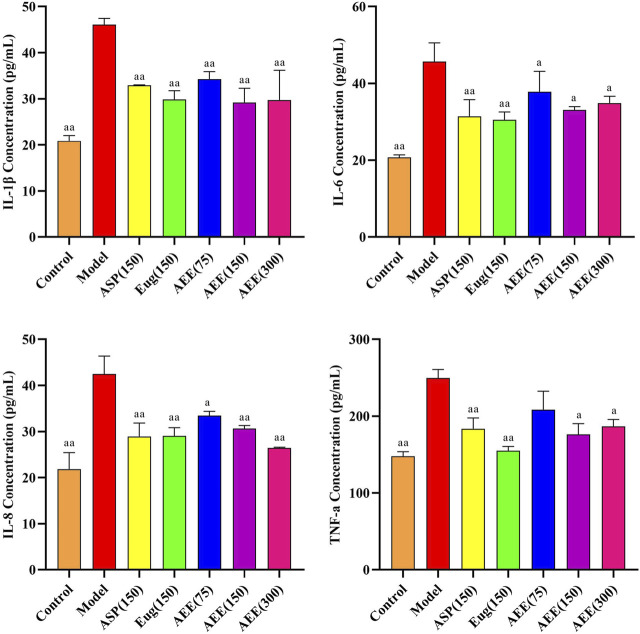
Effect of AEE on IL-1β, IL-6, IL-8, TNF-α in RAW246.7 inflammation model induced by LPS (*n* = 5). Compared with model group: ^a^ *P* < 0.05, ^aa^ *P* < 0.01.

Compared with control group, CCK-8 assay showed that AEE treatment had no inhibitory effect on cell count, and the cell viability of 5, 15, 45, 75, 100, 150, 300, and 350 μM treatment was above 93% (Supplementary Figure S3). In the LPS-induced inflammation model of RAW264.7 cells, the expression of IL-1β, IL-6, IL-8, and TNF-α was significantly higher in the model group compared to the control group (*p* < 0.01) (Figure 4). AEE (150 μM) treatment significantly reduced the expression levels of IL-1β, IL-6, IL-8 and TNF-α compared to the model group (*p* < 0.05 or *p* < 0.01). There was no significant difference in the AEE group compared to the Asp and Eug groups. (*p* > 0.05).

### 3.5 Effects of AEE on NF-κB and MAPK signaling pathways

**FIGURE 5:**
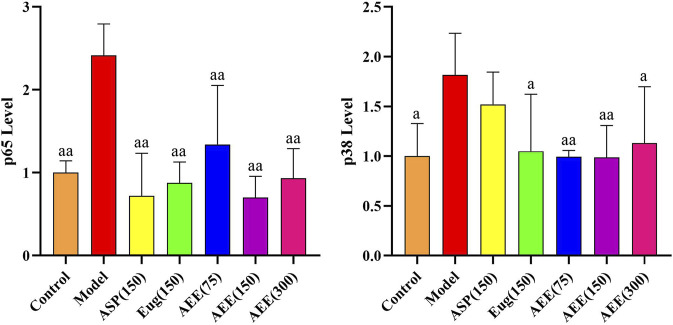
Effects of AEE on NF-κB and MAPK signaling pathways in RAW246.7 cells (*n* = 5). Compared with model group: ^a^ *P* < 0.05, ^aa^ *P* < 0.01.

First, we conducted RT-PCR to assess the mRNA expression of p65 and p38 in RAW264.7 cells. The results demonstrated a significantly lower mRNA expression levels of p65 and p38 in the control group compared to the model group (*p* < 0.01) (Figure 5). AEE (150 μM) caused significant reduction in the mRNA expression levels of p65 and p38 (*p* < 0.05). In regard to p38 level, no difference was found between the model and Asp groups, while Eug and AEE (75, 150, and 300 μM) treatments significantly decreased p38 levels in comparison with the model group. Compared to the Asp and Eug groups, there were no significant differences observed in the AEE three groups in the results of p65 and p38 (*p* > 0.05). Furthermore, no significant differences were found among the three doses of AEE groups (*p* > 0.05).

**FIGURE 6:**
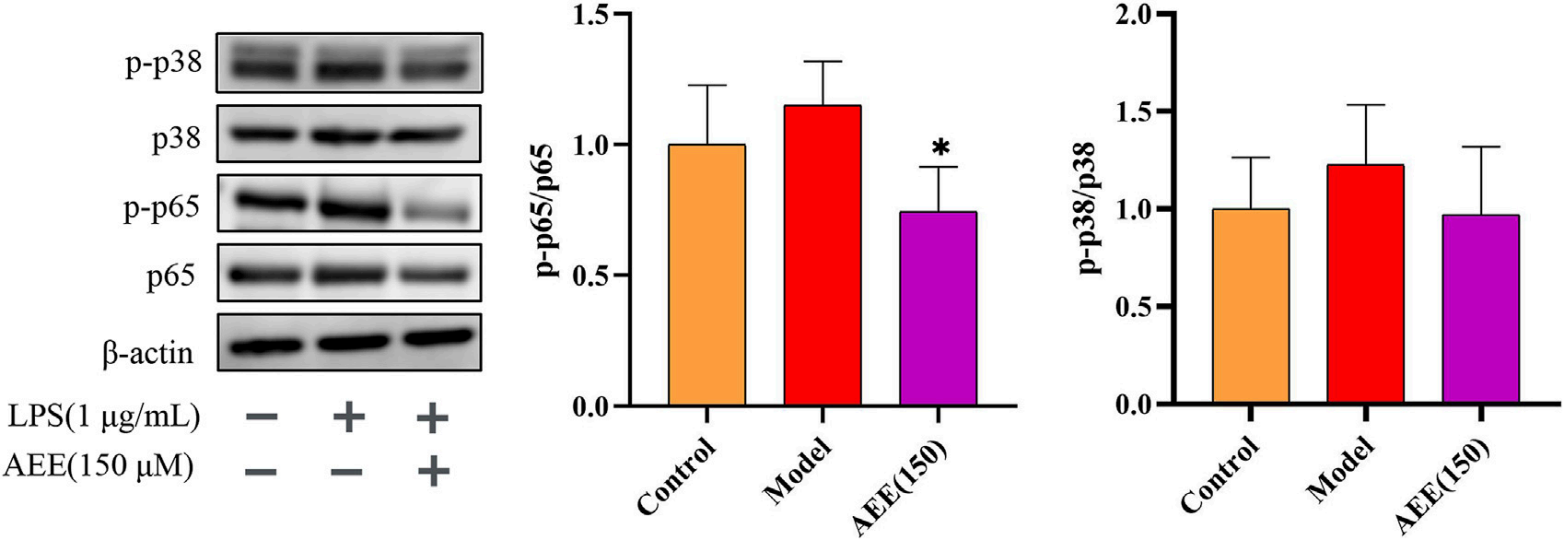
AEE inhibited the activation of NF-κB p65 and p38 MAPK in RAW246.7 cells (*n* = 3). Compared with model group: ^*^ *p* < 0.05.

In order to further investigate the mechanism behind the anti-inflammatory effect of AEE, we conducted Western blot analysis to examine the expression levels of p65 and p38 proteins. In Figure 6, there was no significant difference in the ratios of p-p65/p65 and p-p38/p38 between the control and model groups (*p* > 0.05). Notably, treatment with AEE significantly inhibited the phosphorylation ratio of p65 in comparison to the model group (*p* < 0.05). Similarly, AEE showed inhibition on the ratio of p38 phosphorylation, but the result was not statistically significant (*p* > 0.05).

## 4 Discussion

**FIGURE 7:**
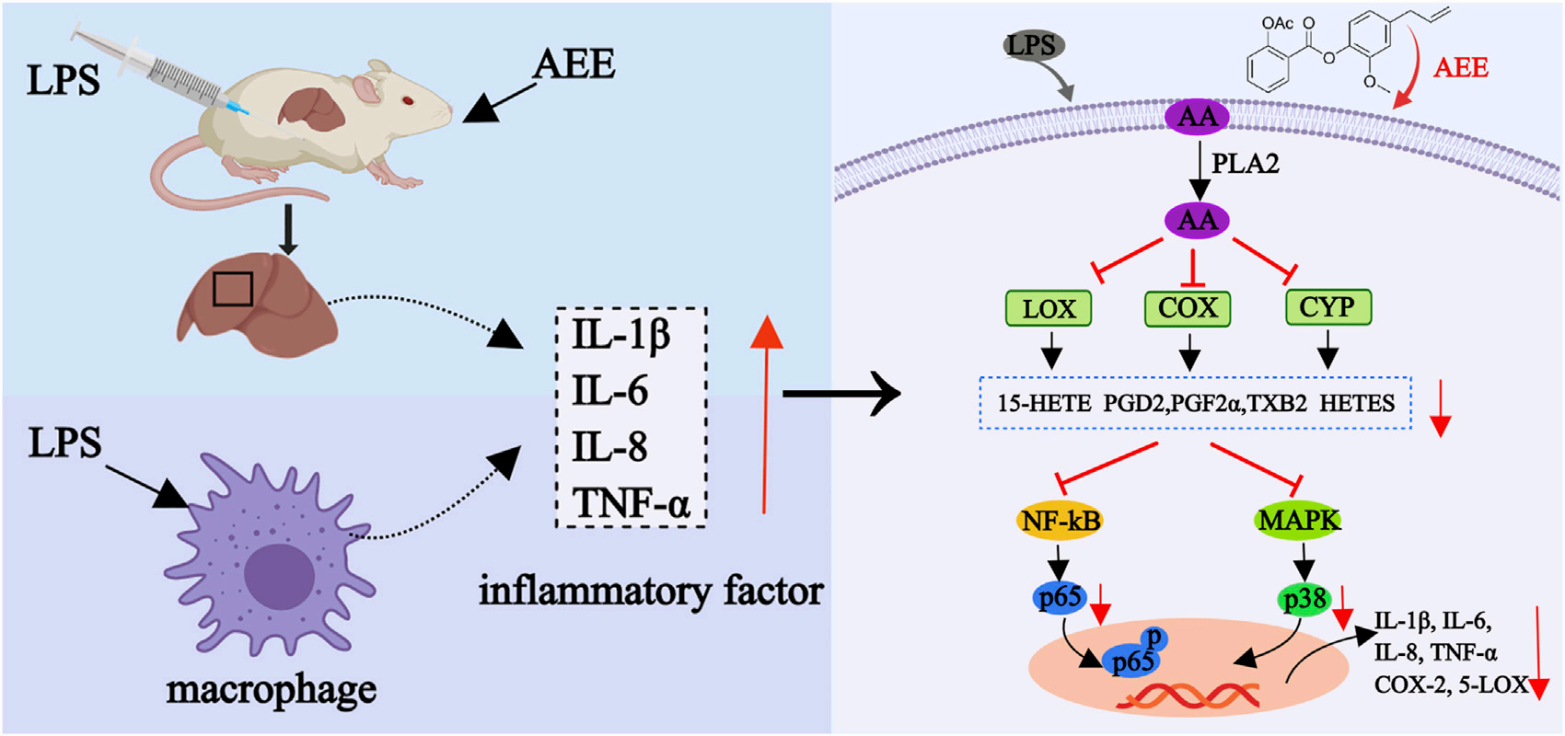
Mechanism of anti-inflammatory action of AEE.

In this study, it was observed that the inflammation triggered by LPS substantially elevated the AA concentration. Additionally, the expression levels of 5-LOX, COX-1, COX-2, PLA2, and CYP450 were upregulated. Moreover, the concentrations of their metabolites such as PGE_2_, TXB_2_, PGF_2α_, PGD_2_, DHA, 15-HETE, 9-HODE and 6-KETO-PGF_1α_ were increased. Subsequently, excess AA may lead to an inflammatory response through the activation of NF-κB p56 and MAPK p38. The results revealed that the AEE decreased the levels of inflammatory factors including TNF-α, IL-8, IL-6, and IL-1β. Furthermore, AEE treatment led to a trend of reduction in the concentrations of AA and its metabolites such as TXB_2_, PGF_2α_, PGD_2,_ DHA, 15-HETE, 9-HODE, and 6-KETO-PGF_1α_. As the main inflammatory mediator, the level of PGE_2_ was not reduced by AEE treatment. COX catalyzes the conversion of AA to PGG_2_ and PGH_2_ (Das et al., 2007). Then, PGE_2_ is synthesized from PGH_2_ under the action of microsome PGE synthetase-1 (mPGES-1), mPGES-2 and cytoplasmic PGE synthetase (Zhang et al., 2022). In this study, although COX was found to be downregulated in AEEM group, the regulating PGE synthetases were not detected, which might be the possible reason that there was no significant change in PGE_2_. Additionally, AEE decreased the ratios of p-p65/p65 and p-p38/p38 (Figure 7).

The quantity and ratio of blood cells serve as indicators of inflammation and prognosis in numerous diseases, reflecting the health status of the body. As an important indicator of immune function and body condition, the WBC is involved in important functions such as the immune response to disease and endocrine function. Zucoloto *et al.* used LPS to induce an inflammatory in mice and confirmed that probucol suppressed the total leukocyte count in response to inflammation (Zucoloto et al., 2017). In this study, AEE treatment significantly decreased the leukocyte count in mice, indicating that AEE effectively mitigated the inflammatory response induced by LPS.

In many inflammatory diseases, inflammatory factors become abnormal, and are important markers for the diagnosis of various diseases. Therefore, the downregulation of inflammatory factor expression plays a crucial role in disease management and prevention (Tuomisto et al., 2019). LPS is the main component of the cell wall of Gram-negative bacteria, which is frequently used as the inflammatory inducer (Liu et al., 2017). *In vivo*, LPS predominantly binds to toll-like receptors located on the surface of macrophages, triggering downstream signaling pathways to stimulate the production of various inflammatory factors, chemokines, and reactive oxygen species (Ma et al., 2018). Previously, Xie *et al.* used 1 μg/mL of LPS to induce inflammation and oxidative stress model in RAW264.7 cells (Xie et al., 2019). Similarly, LPS (5 mg/kg) was used to induce septic lung injury and systemic inflammation in mice (Hwang et al., 2019). In present study, the levels of inflammatory factors IL-1β, IL-6, IL-8, and TNF-α showed a significant increase following LPS stimulation. This demonstrated that the inflammation model was successfully modeled and aligned with the findings of a previous study (Moon et al., 2019). The levels of IL-1β, IL-6, IL-8, and TNF-α were decreased after treatment with AEE, indicating the protective effects of AEE on inflammation response.

The enzymes COX, LOX, and CYP450 are responsible for catalyzing the synthesis of AA eicosanoids, which play a crucial role in inflammatory diseases (Werz, 2002). Inhibition of the COX and LOX can reduce the production of PGs and LTs which are two important inflammatory mediators. In this experiment, the expression levels of 5-LOX, COX-1, COX-2, PLA2, and CYP450 were increased in the LPS group, confirming the involvement of COX, LOX, and CYP450 in the inflammatory response process. Asp and Eug are non-selective inhibitors of COX, exhibiting inhibitory activity against both COX-1 and COX-2 *in vitro and in vivo* (Liu et al., 2018; Wang et al., 2022). Singh *et al.* demonstrated that Asp specifically binds to PLA2 and has inhibitory effect on it (Singh et al., 2008). Wen *et al.* showed that long-term administration of Asp resulted in an increase in the number of liver mitochondria and the levels of glutamic pyruvic transaminase and glutamic oxaloacetic transaminase, affecting the metabolic activity of CYP450 (Wen et al., 2018). It was found that AEE downregulated the mRNA levels of 5-LOX, COX-1, COX-2, PLA2, and CYP450 in the liver of the mice with inflammation. However, the effects of AEE on these enzymes are needed to validated by more studies such as WB and enzyme inhibition experiments. The dual inhibitory effect of AEE on COX and LOX may be one of the mechanisms by which AEE suppresses the inflammatory response induced by LPS. The experimental results suggest that AEE may be similar to the anti-inflammatory mechanism of Asp. PLA2 plays a role in catalyzing the release of AA from the phospholipid pool in cell membranes. The RT-PCR results revealed that AEE reduced the expression of PLA2 in the liver of mice. Therefore, we proceeded to investigate the AA metabolites in order to explore the anti-inflammatory pathway of AEE through targeted metabolomics.

As important inflammatory biomarkers, AA and its metabolites are widely involved in immune and inflammatory responses *in vivo* (Romano et al., 2015). The liver is the main site of AA metabolism, which is closely related with inflammation (Aslan et al., 2014). Accumulated monocytes or macrophages in the liver are sensitized in the presence of pathogenic factors. In this situation, the AA metabolism will be disturbed in these inflammatory cells, resulting in the release of inflammation mediators such as PGs, LTs and TXs. In previous studies, the AA metabolism in the liver was explored to explore the action mechanism of drug on inflammation (Yang et al., 2021; Wang et al., 2023). The important AA derived mediators and regulators include PGs, TXs, leukotrienes (LTs), LXs, hydroper oxy-eicosatetraenoic acid (HPETE), and hydroxy-eicosatetraenoic acid (HETE) (Peebles and Aronica, 2019). Previous studies have provided evidence that increased levels of PGD_2_ and TXA_2_ contributed to the production of pro-inflammatory cytokines such as IL-4, IL-5, and LTB_4_ (Wang et al., 2021). In this study, we employed LC-MS/MS to measure the concentration of AA metabolites in mouse liver tissues, and found that AA, PGD_2_, and TXB_2_ were increased in the model group. Rossi *et al.* demonstrated that decreased levels of 6-KETO-PGF_1α_ in LPS-induced RAW264.7 cells might inhibit PG biosynthesis by interfering with AA release. (Rossi et al., 2010). Hamid *et al.* showed that Asp reduced the levels of the inflammatory cytokine TNFα and TXB_2_ in a model of LPS-induced lung inflammation (Hamid et al., 2017). In this study, AEE treatment resulted in significant reductions in AA concentrations. Notably, AEE also decreased the concentrations of 6-KETO-PGF_1α_, PGD_2_, PGF_2α_, and TXB_2_. These findings indicate that AEE may inhibit the LPS-induced inflammatory response by modulating the AA metabolic pathway.

The NF-κB and MAPK pathways are known as downstream inflammatory pathways of AA metabolism (Shoeb et al., 2011). NF-κB plays an important role in regulating the transcription of numerous genes involved in immunity, inflammation, and apoptosis (Longpre et al., 2006). Previous studies have demonstrated that the p38 MAPK and NF-κB signaling pathways are activated in response to abnormal levels of inflammatory cells. When NF-κB expression is inhibited, the occurrence of inflammation may be improved (Lu et al., 2021). The p38 MAPK pathway has been shown to positively regulate the expression of various genes associated with inflammation, including TNF-α, IL-1β, IL-6, and IL-8 (Manthey et al., 1998). In addition, Murakami *et al.* suggested that compounds related to Eug may possess antioxidant and anti-inflammatory effects through the inhibition of NF-κB activation (Murakami et al., 2017). The results from RT-PCR and Western blot analysis demonstrated that AEE treatment decreased the relative mRNA expression levels of p65 and p38, and the ratios of p-p65/p65 and p-p38/p38. These findings suggested that AEE might exert its anti-inflammatory effects by down-regulating the NF-κB and MAPK signaling pathways. It was reported that Asp exerted the anti-inflammatory effects mainly through irreversible binding to COX, inhibiting the development of inflammation (Poorani et al., 2016). In addition, Asp inhibits inflammation by regulating NF-κB signaling pathway (Tung et al., 2022). Notably, both AEE and Asp had inhibitory effects on COX and NF-κB signaling pathway, indicating that they might have the same pharmacological mechanism on inflammation.

Additionally, it is crucial to notice that the current study has certain limitations. Firstly, the number of AA metabolites tested in the experiment was few. In previous studies, 19 metabolites were detected (Wang et al., 2018). However, in this study, only 11 metabolites were detected in liver. The systemic biosynthesis of AA metabolites in other biological matrices such as urine and blood should be assessed, which benefit to gain insight into the mechanism of action of AEE on inflammation. Secondly, the sample size in the AA target metabolomics study was small. The complicated effect of AEE on AA metabolic pathway needs to be explored by large number of samples and more studies. For example, the levels of biomarkers such as TXB_2_ and PGE_2_ in whole blood should be analyzed to reflect the effects of AEE on the activities of COX-1 and COX-2. The signaling pathway can be further analyzed in depth through the methods such as knockdown or overexpression assays. Thirdly, the difference on the action mechanism between Asp and AEE on inflammation should be explored in further studies.

## 5 Conclusion

In summary, AEE significantly inhibited the LPS-induced inflammatory response by reducing the levels of inflammatory metabolites in the AA metabolic pathway. In addition, AEE caused a reduction in the expression of p65 and p38 within the NF-κB and MAPK signaling pathways. These findings establish an experimental foundation for the potential clinical use of AEE in diseases associated with inflammation.

## Data Availability

The original contributions presented in the study are included in the article/Supplementary Material, further inquiries can be directed to the corresponding authors.
