# Supplementary material for: Aspirin eugenol ester ameliorates LPS-induced inflammatory responses in RAW264.7 cells and mice
Source: Front Pharmacol. 2023 Aug 29;14:1220780. doi: 10.3389/fphar.2023.1220780 (PMC10495573; doi:10.3389/fphar.2023.1220780)
Supplement: Supplementary file 1 [file Presentation1.PDF]

Supplementary Material

AEE Inhibits Lipopolysaccharide-induced Inflammatory Responses via the Arachidonic Acid Metabolic Pathway

Xu Liu[1#], Qi Tao[2#], Youming Shen[3], Xiwang Liu[2], Yajun Yang[2], Ning Ma[1*], Jianyong Li[2*]

*** Correspondence:** Ning Ma, Address: No.2596, Lekai South Street, Baoding 071000, China.

E-mail: maning9618@163.com; Phone: 0086-0312-7520270

Jianyong Li, Address: No.335, Jiangouyan, Qilihe district, Lanzhou 730050, China.

E-mail: lijy1971@163.com; Telephone/Fax: 0086-0931-2115290

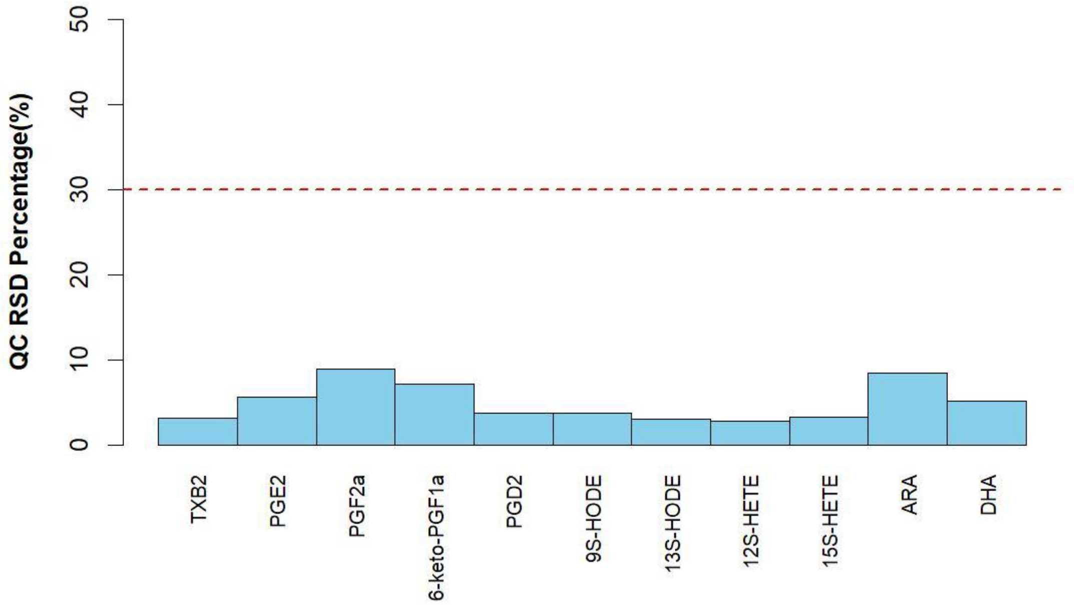

Figure S1 Relative standard deviation distribution of quality control samples.

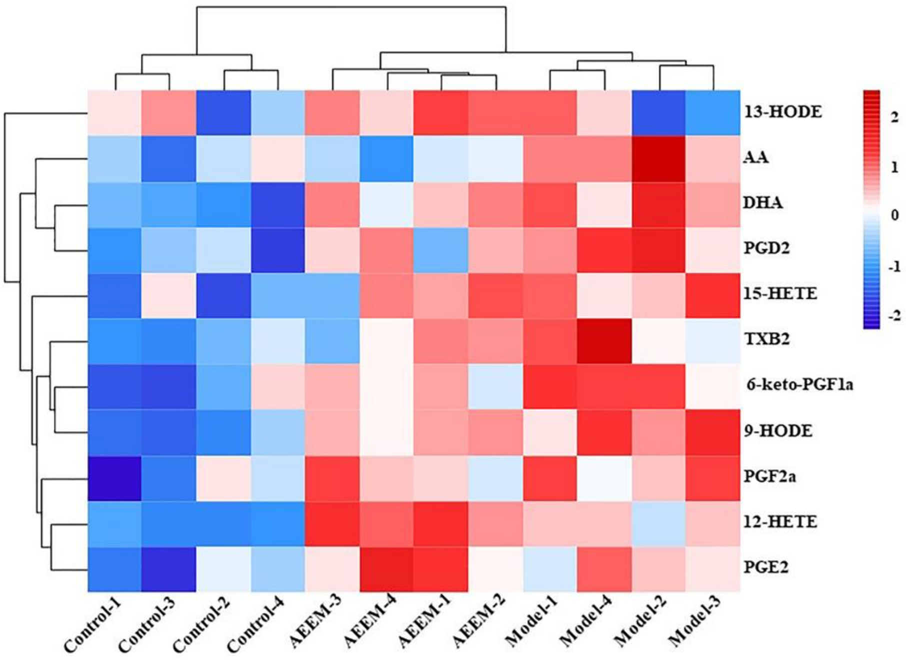

Figure S2 Hierarchical cluster analysis (heat map) of AA metabolites (n = 4). The color bar representing the Z-score, red represents high expression, and blue represents low expression.

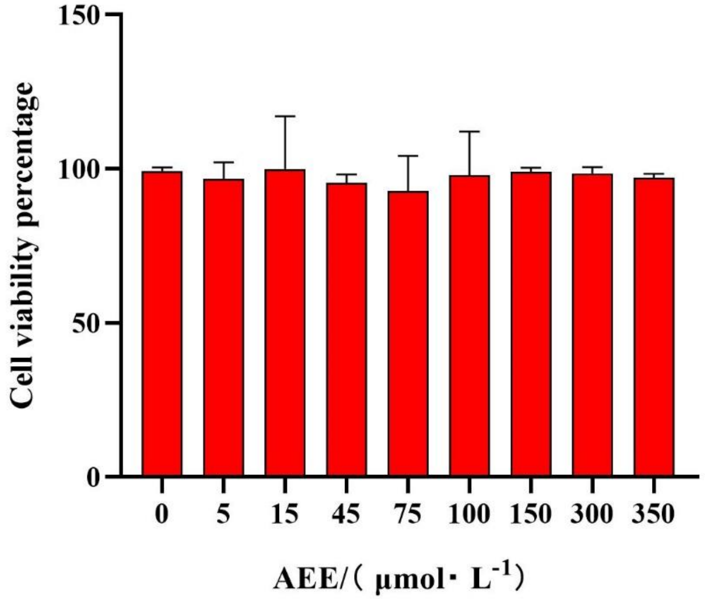

Figure S3 The effect of AEE on the viability of RAW264.7 cells (n=5)**.**

Table S1 Standard curve results for AA metabolites

Component Name	Mass Info	Retention Time (min)	Linear	R
TXB_2	369.2/169.0	2.26	y=0.01526x+0.05919	0.99986
PGE_2	351.2/271.3	2.93	y=0.01154x+0.00335	0.99973
$PGF_{2\alpha}$	353.1/309.3	2.60	y=0.02016x+0.01568	0.99959
6-keto-$PGF_{1\alpha}$	369.3/163.0	1.16	y=0.01243x+0.01283	0.99989
PGD_2	351.2/271.3	2.75	y=0.04039x+0.02172	0.99995
9-HODE	295.1/171.1	5.63	y=0.01000x+0.02284	0.99896
13-HODE	295.0/195.0	5.58	0.01527x +0.08743	0.99986
12-HETE	319.1/179.0	5.97	0.01454x+-0.03290	0.99977
15-HETE	319.1/219.0	5.73	0.00304x+-0.00213	0.99994
AA	303.2/259.2	7.80	0.00282x +0.00365	0.99900
DHA	327.1/229.2	7.64	0.00151x+0.00188	0.99985

Abbreviation: $TXB_{2,}$ thromboxane $B_{2;}$ $PGE_{2,}$ prostaglandin E_2; $PGF_{2\alpha,}$ prostaglandin $F_{2\alpha;}$ 6-keto-$PGF_{1\alpha,}$ 6-keto-prostaglandin $F_{1\alpha}$; $PGD_{2,}$ prostaglandin D_2; 9-HODE, 9-hydroxyoctadecadienoic acid; 13-HODE, 13-hydroxyoctadecadienoic acid; 12-HETE, 12-hydroxyeicosatetraenoic acid; 15-HETE, 15-hydroxyeicosatetraenoic acid; AA, arachidonic acid; DHA, docosahexaenoic acid.

Table S2 Sequence of primers used in RT-PCR

Gene	Primer sequences（5’-3’）
COX-1	F: GCCCTTCAATGAATACCGAAAG<br>R: GGGTAGAACTCTAAAGCATCGA
COX-2	F: ATTCCAAACCAGCAGACTCATA<br>R: CTTGAGTTTGAAGTGGTAACCG
CYP450	F: TCATGAAGCACAGTCACTACAT<br>R: AAACTTCCACCATTCGAACAAG
PLA2	F: CAACTGGAGGAAAAAGACTGTG<br>R: TTGTAAGTCCAGAGGTTTCTCC
5-LOX	F: GGCGAGATCTACCTAGTCAAAA<br>R: GATGTGAATTTGGTCATCTCGG
p65	F: CTGGCGCAGAAGTTAGGTCT<br>R: GCTGCCTGGATCACTTCAATG
p38	F: AGGACAGGAACTCACACAGGGTAG<br>R: AGCAAGCCAGTAAGCAGCATCAG
β-actin	F: AACTCCATCATGAAGTGTGA<br>R: ACTCCTGCTTGCTGATCCAC
