# Supplementary material for: Aspirin eugenol ester ameliorates LPS-induced inflammatory responses in RAW264.7 cells and mice
Source: Front Pharmacol. 2023 Aug 29;14:1220780. doi: 10.3389/fphar.2023.1220780 (PMC10495573; doi:10.3389/fphar.2023.1220780)
Supplement: Supplementary file 2 [file Presentation2.PDF]

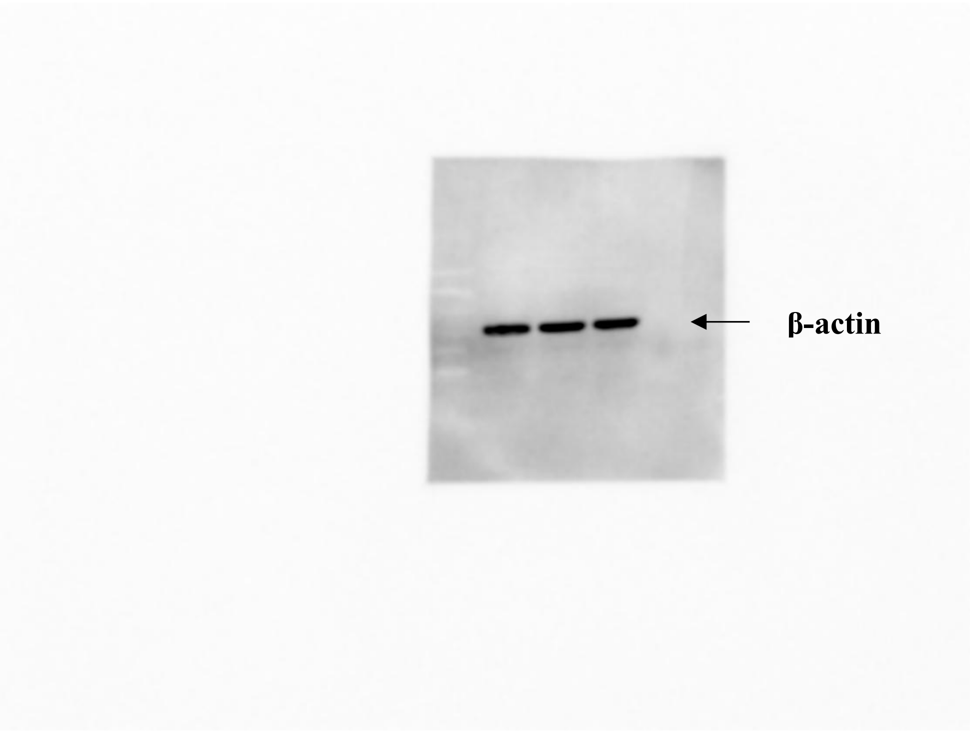

Fig.7 AEE inhibited the activation of NF-κB p65 and p38 MAPK in RAW246.7 cells.

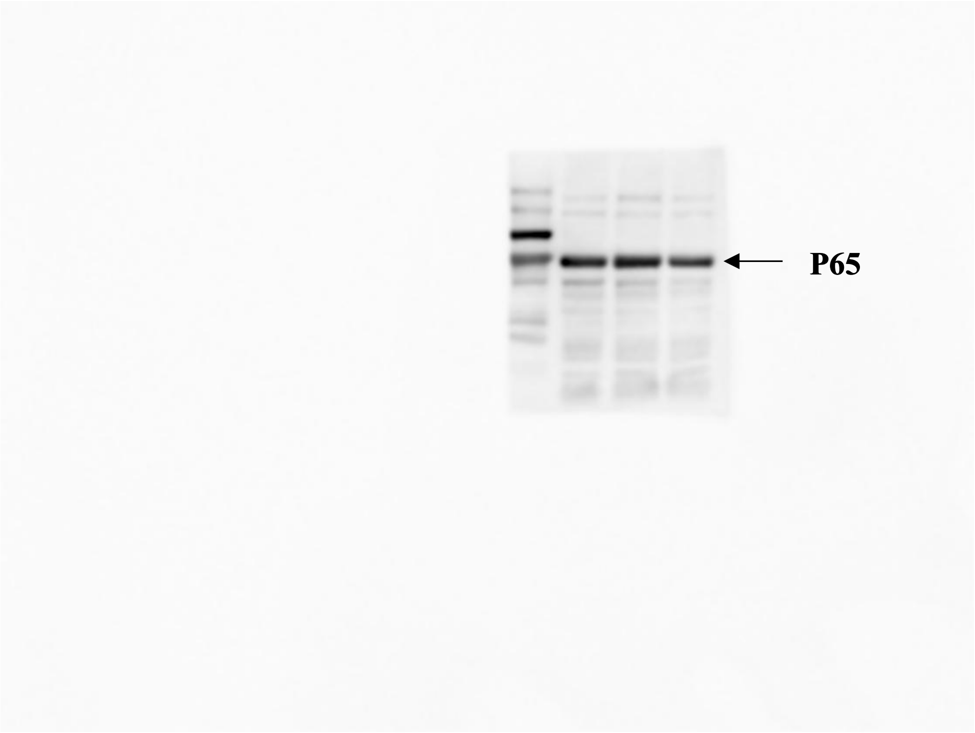

Fig.7 AEE inhibited the activation of NF-κB p65 and p38 MAPK in RAW246.7 cells.

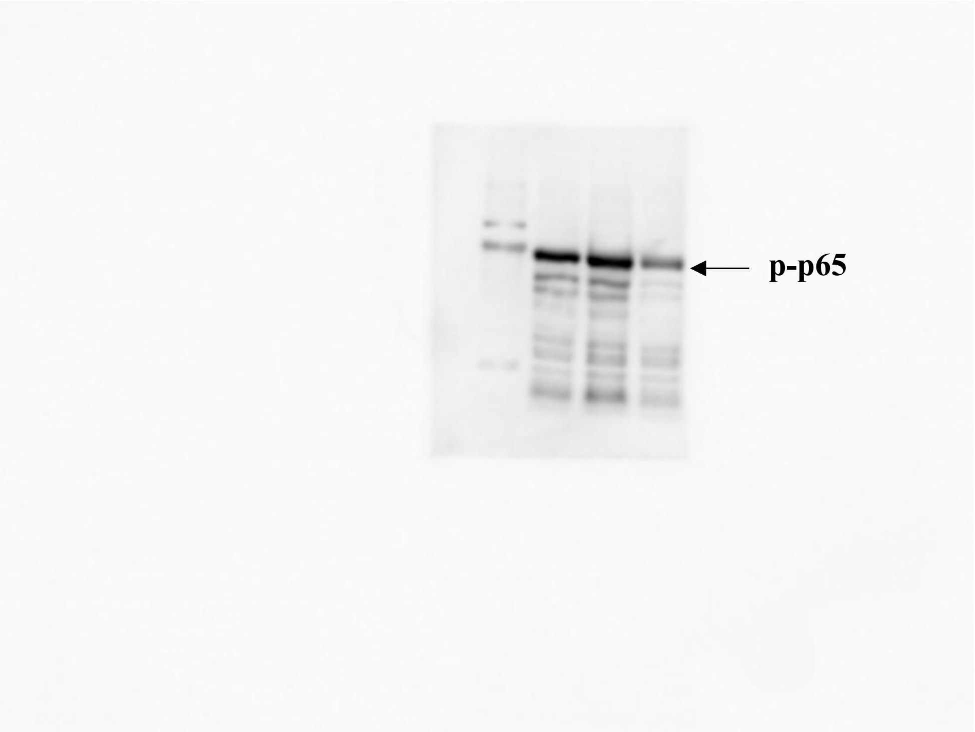

Fig.7 AEE inhibited the activation of NF-κB p65 and p38 MAPK in RAW246.7 cells.

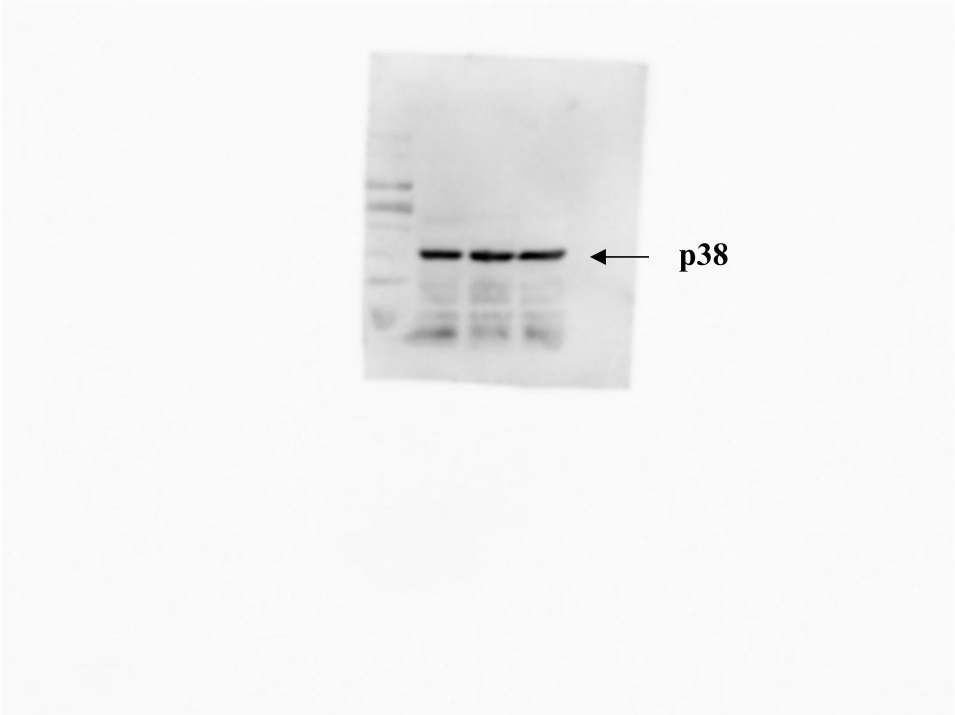

Fig.7 AEE inhibited the activation of NF-κB p65 and p38 MAPK in RAW246.7 cells.

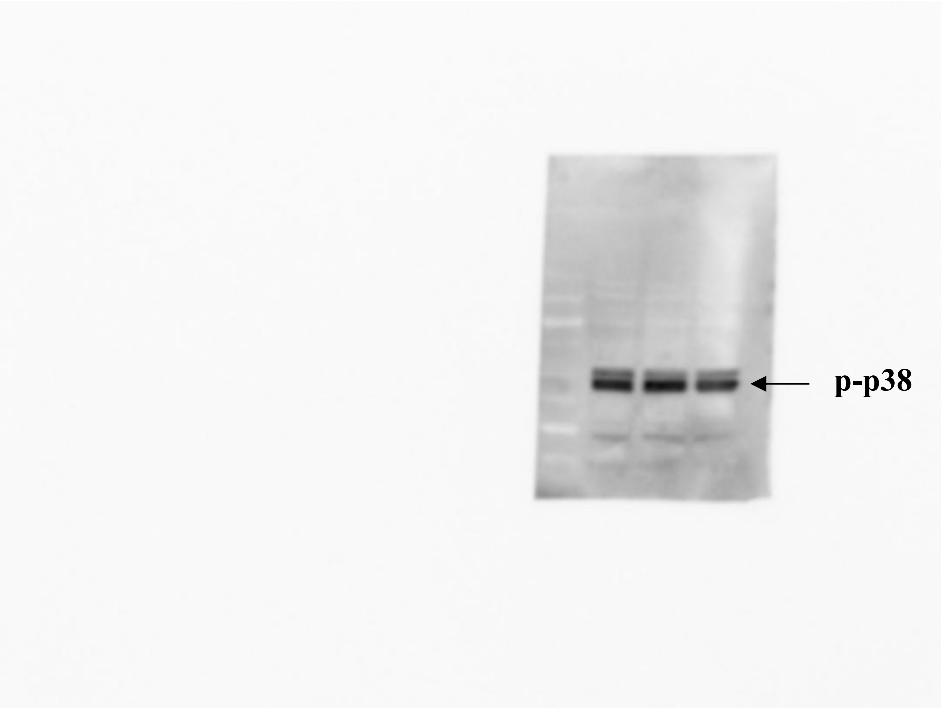

Fig.7 AEE inhibited the activation of NF-κB p65 and p38 MAPK in RAW246.7 cells.
